# Quercetin Attenuates Nitroglycerin-Induced Migraine Headaches by Inhibiting Oxidative Stress and Inflammatory Mediators

**DOI:** 10.3390/nu14224871

**Published:** 2022-11-17

**Authors:** Ahmed I. Foudah, Sushma Devi, Mohammed H. Alqarni, Aftab Alam, Mohammad Ayman Salkini, Manish Kumar, Husam Saad Almalki

**Affiliations:** 1 Department of Pharmacognosy, College of Pharmacy, Prince Sattam Bin Abdulaziz University, P.O. Box 173, Al-Kharj 11942, Saudi Arabia; 2 Chitkara College of Pharmacy, Chitkara University, Rajpura 140401, India; 3 Department of Neurosurgery, College of Medicine, Penn State Health Milton S. Hershey Medical Center, The Pennsylvania State University, State College, PA 17033-0850, USA

**Keywords:** migraine, quercetin, locomotor behavior, nitroglycerine, antimigraine

## Abstract

This study aimed to investigate the antimigraine potential of quercetin in migraine pain induced by nitroglycerin (NTG), 10 mg/kg, intraperitoneal injection in rats. Quercetin was administered orally for 1 week, and behavioral parameters associated with pain were assessed 30 min after NTG injection. At the end of the study, the rats were killed so that immunohistochemical examination of their brains could be performed. The time and frequency of rearing and sniffing in the category of exploratory behavior, walking in the category of locomotor behavior, and total time spent in the light chamber were reduced in the disease control group compared with the normal group during the assessment of behavioral parameters. Pathologic migraine criteria, such as increased levels of calcitonin gene-related peptide and increased release of c-fos cells, were more prominent in the caudal nucleus triceminalis of the NTG control group. In the treatment groups, behavioral and pathological measures were less severe after pretreatment with quercetin at doses of 250 and 500 mg/kg. Therefore, it was concluded that quercetin improved the pain behavior of migraine patients in the NTG-induced migraine rat model. Quercetin is thought to have antimigraine effects due to its antioxidant and anti-inflammatory potential. Quercetin may therefore be a novel agent that can treat or prevent migraine pain and associated avoidance behaviors.

## 1. Introduction

Most often, any type of pain is due to inflammation or nerve damage. Migraine is a recurrent neurological pain disorder that affects the health, social, and economic situation of patients. According to epidemiological reports, migraine affects 1 in 10 people worldwide, and its incidence continues to increase inconspicuously [1]. In the Global Burden of Diseases 2016, migraine was reported to be the sixth most common disease among 328 diseases and harms. In addition, migraine was ranked as the second most common disease in which life is lived with disability [2]. Migraine is defined according to the International Classification of Headache Disorders as a “recurrent headache disorder manifested in attacks lasting 4–72 h.” Migraine is characterized by moderate to severe throbbing or pulsating pain. The pain may be unilateral, may have a pulsatile quality, and may interfere with physical activity. In addition to the typical features, other symptoms may include nausea, fatigue, photophobia, or phonophobia [3].

All over the world, pain treatment with classical drugs (NSAIDs) is a challenge. This is because such treatments are associated with many side effects and are iatrogenic. In clinical trials, these NSAIDs showed numerous side effects and led to an increase in gastrointestinal ulcers, as well as cardiac and renal diseases [4,5]. Thus, there is a need for analgesic drugs with fewer or minimal side effects. Opioids are used to relieve pain in patients, depending on the severity of pain. Similar to NSAIDS, they are associated with numerous side effects, such as drowsiness, addiction, constipation, increasing changes in respiratory disorders, and morbidity [6]. However, the use of opioids in the treatment of various types of pain remains problematic due to symptomatic side effects. In addition, agents such as corticosteroids, immunobiological agents, opioids, etc., are associated with immunosuppression [7]. Therefore, researchers are increasingly searching for natural agents that are associated with negligible side effects. Therefore, over the years, more attention has been paid to phytoconstituents such as flavonoids, terpenoids, alkaloids, etc. Flavonoids are a highly diversified and multi-substituted subgroup of polyphenolic compounds, whose basic structural representative is the flavan core [8].

Quercetin is a rich flavonol found in plants, fruits, onions, vegetables, bark, tea, and so on. It is a powdery, yellowish compound that dissolves completely in alcohol and lipids. Quercetin is not produced/formed in the human body [9,10]. There is an extensive literature on the pharmacological effects of quercetin, such as. anticancer, antitumor [11], immunomodulatory [12], antioxidant [13], anti-inflammatory [14], cardioprotective [15], antiatherosclerotic [16], antidiabetic [17], antibacterial [18], antiviral, antiprotozoal, antimicrobial [19], hepatoprotective [20], antihypertensive [21], antineurodegenerative [22], and so on. Quercetin showed efficacy against inflammation due to its potent antioxidant activity. The anti-inflammatory, antiamyloidogenic and antioxidant activities are helpful in the treatment of cardiovascular diseases and cancer [17,23,24,25]. According to one report, quercetin showed cardioprotective activity in a 6-week study by lowering oxidized low-density lipoprotein levels and blood pressure [24]. In another study, quercetin was found to be effective against diastolic, systolic, and arterial blood pressure and showed excellent antihypertensive activity in 4 weeks of treatment [21]. Similarly, in type 2 diabetes, efficacy was observed in regulating hypertension with 10 weeks of treatment [17]. Therefore, it was found in the literature that quercetin also has a preventive effect against cardiovascular risk factors and may be an additional therapeutic approach [16]. In addition, quercetin improved rheumatoid arthritis symptoms such as stiffness and pain, and also lowered C-reactive protein and TNF-α plasma levels in an 8-week study [26]. Quercetin ameliorates hyperinflammation associated with X-linked apoptosis inhibitor deficiency and inhibitory effects on β-secretase in AD [27]. Quercetin interfered with nitric oxide and cyclooxygenase pathways and blocked calcium channels to induce basilar artery relaxation in an in vitro study [28]. Therefore, quercetin was selected in the present study to investigate its effect in migraine. Moreover, it can be used as a potential agent in the treatment of migraine. Systemic administration of NTG induces migraine symptoms in animals similar to those in humans. Thus, it is the second most common experimental animal model for migraine [25]. Therefore, the present study aimed to investigate the antimigraine potential and mechanism of action of quercetin using a known NTG-induced migraine in rats.

## 2. Materials and Methods

### 2.1. Chemicals

Quercetin (gift sample from MyoFord Pharma, Shimla, India), nitroglycerin, normal saline, and sumatriptan (Sigma Aldrich, Bengaluru, India). All chemicals and kits used in this study were laboratory grade.

### 2.2. Animals

Sprague Dawley rats weighing 210–260 g were used in the present study, as shown in Table 1. The Standing Committee on Bioethic Research (SCBR-024-2022) of Prince Sattam Bin Abdulaziz College, Al-Kharj, Ministry of Education, Kingdom of Saudi Arabia, approved the studies. Rats were maintained in controlled light–dark cycles and humidity at room temperature of 25 °C ± 1 °C. The rats had free access to food and water.

### 2.3. Experimental Design

The rats were divided into different groups and each group contained 6 rats. Quercetin (250 and 500 mg/kg) and sumatriptan 50 mg/kg were administered orally daily for 7 days at the same time. NTG (10 mg/kg) was administered intraperitoneally in the different groups on the eighth day to induce an acute migraine attack. 

Then, rats were exposed to a sequence of behavioral tests after induction of migraine [29,30,31]. 

Module 1—Acute spontaneous migraine-like behavior recognition test.

Module 2—Light-aversive behavior in light/dark test.

Module 3—Rough and smooth surface apparatus test.

Module 4—Open-field test.

Rats were sacrificed, and their trigeminal nucleus caudalis (TNC) was stored in 10% formalin solution for immunohistochemical staining. Then, the calcitonin-gene-related peptide (CGRP) and the amount of positive staining were analyzed.

**Figure 1:**
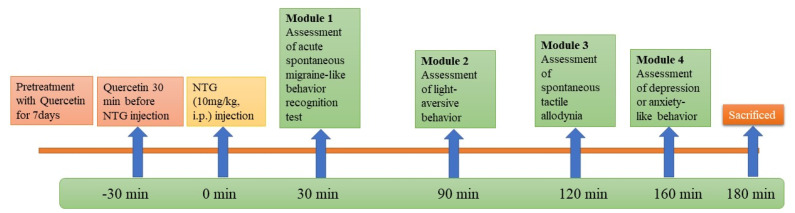
Workflow for animal study.

The experimental procedure is shown in Figure 1.

#### 2.3.1. Module 1-Analysis of Migraine-like Behavior

After 30 min of NTG administration, the rat was placed in a transparent box, and migraine-like behaviors were exhibited, such as running and jumping (locomotor behavior), retrieving and sniffing (exploratory behavior), immobile posture or sleeping (resting behavior), twitching (freezing behavior), and preening face or body (grooming behavior). For the above activities, the rat spent the entire time performing each behavior, except for the twitching behavior. For the twitching behavior, only the frequency was recorded [31].

#### 2.3.2. Module 2-Analysis of Light-Aversive Behavior

After 90 min of NTG administration, rats were placed in a light/dark box and their light-aversive behavior was analyzed. The light/dark box consists of two identical chambers (32 × 32 × 25 cm^3^). One of the two chambers is illuminated at 1000 lx, and the other has walls painted black and a lid to observe the activity of the rats. There is a small opening between the two chambers through which the rats can move freely between the chambers. First, the rat was positioned in the center of the light chamber, and then its movement in the chambers was observed for 10 min. When assessing light-shy behavior, the total distance and total time the rats spent in the light chamber and the number of transitions between the two chambers were noted [30].

#### 2.3.3. Module 3-Analysis of Spontaneous Tactile Allodynia

After 120 min of NTG administration, rats were placed in a rough/smooth surface apparatus and assessed for spontaneous tactile allodynia. The apparatus chamber was made of clear acrylic and had two identical areas with rough and smooth bottoms (made of sandpaper). Initially, the rat was positioned in the center of the chamber and then allowed to move freely over the surfaces for 5 min. When analyzing spontaneous tactile allodynia behavior, the parameters, i.e., total time spent on the rough and smooth surfaces, were noted [31,32].

#### 2.3.4. Module 4-Analysis of Anxiety or Depression Like Behavior

After 160 min of NTG administration, the anxiety- or depression-like behaviors of the rats were assessed. The rat was placed in an open field apparatus (60 × 60 × 60 cm^3^) with a black wooden floor. Initially, the rat was placed in the center of the apparatus and allowed to freely explore the central area (30 cm × 30 cm) for 5 min. The total distance traveled and the total time the rat spent in the central area served as the anxiety or depression index [30,31].

#### 2.3.5. Immunohistochemistry Staining Study

The modified method of Liao et al. (2019) was used for immunohistochemical analysis. After 180 min of NTG administration, three rats from each group were sacrificed. The brains of the rats were removed and stored in 10% formalin for IHC analysis. Immunohistochemical staining of TNC was performed with the assistance of a pathological expert and followed Max Labs protocols for paraffin-embedded sections. After slide preparation, sections were counterstained with hematoxylin for 5 min and then rinsed 2–3 times with double-distilled water. Subsequently, the positive C-Fos and CGRP immunostainings were detected under the light microscope. The number of positive staining was evaluated by experts [31].

### 2.4. Statistical Analysis

All data were generated as mean ± SEM. All data were analyzed by one-way analysis ANOVA, followed by Tukey’s post hoc test. A *p* value of <0.05 was considered statistically significant, and Graphpad Prism software was used.

## 3. Results

### 3.1. Effect of Quercetin on Acute Spontaneous Migraine-like Behavior

#### 3.1.1. Effect of Quercetin on Rearing Up and Sniffing (Exploratory Behavior)

**Figure 2:**
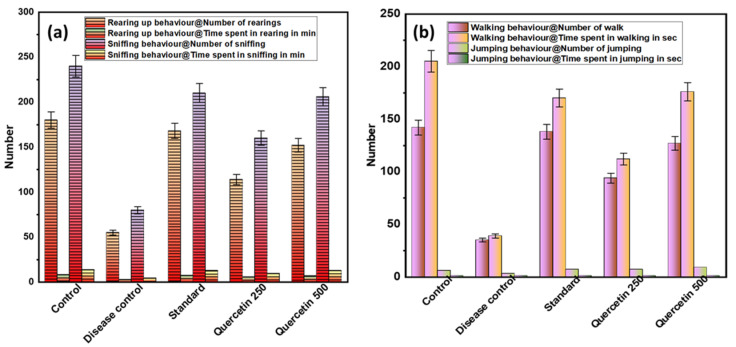
(**a**) Effect of Quercetin on rearing up and sniffing (exploratory behavior); (**b**) effect of Quercetin on walking and jumping (locomotor behavior).

To investigate acute spontaneous migraine-like behavior in rats, the behavioral parameters, i.e., frequency and duration of rearing and sniffing, were measured (see Figure 2a). The disease control group showed less rearing behavior than the normal control group (*p* < 0.001). The quercetin-treated groups showed significantly more rearing behavior compared to the control group. Similarly, there was sniffing behavior in the disease control group compared to the normal control group (*p* < 0.001). Quercetin-treated rats were more involved in sniffing behavior. In the disease control group, the total time spent rearing and sniffing was significantly lower than in the normal control group (*p* < 0.001). The maximum time spent rearing and sniffing was found in the quercetin 500 mg/kg group (*p* < 0.05).

#### 3.1.2. Effect of Quercetin on Walking and Jumping (Locomotor Behavior)

The disease control group showed a lower number of walking and jumping activities and less locomotor behavior compared with the normal control group (*p* < 0.01), as shown in Figure 2b. The quercetin-treated groups showed more walking and jumping activities compared to the control group. In the quercetin-treated group (500 mg/kg), the maximum total walking time was 176 s. The disease control group had a minimum walking time of 39 s compared to the normal control group (*p* < 0.01). When analyzing the total time spent jumping, no statistically significant difference was found between the groups.

#### 3.1.3. Effect of Quercetin on Freezing Behavior (Twitching)

**Figure 3:**
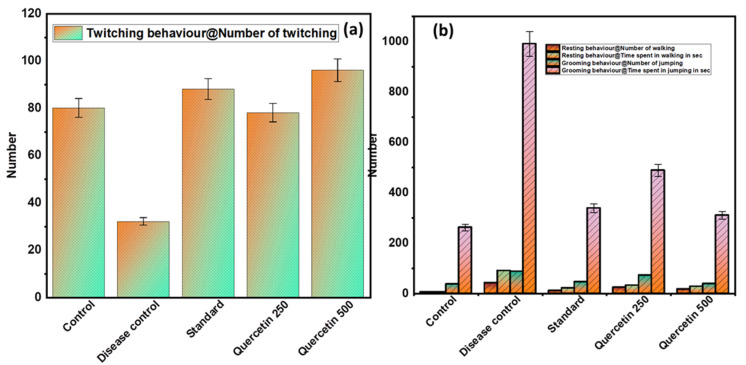
(**a**) Effect of Quercetin on freezing behavior (twitching); (**b**) effect of Quercetin on immobile posture or sleeping (resting and grooming behavior).

The disease control group showed a minimal number of twitches compared to the normal control group and was found to be significant (*p* > 0.01). The quercetin groups caused a significantly higher number of twitches compared to the disease control group (*p* < 0.01) (see Figure 3a).

#### 3.1.4. Effect of Quercetin on Immobile Posture or Sleeping (Resting and Grooming Behavior)

The disease control group showed statistically (*p* < 0.01) greater frequency of resting behaviors, including immobile posture or sleeping, compared with the normal control group (see Figure 3b). The quercetin-treated groups were less likely to adopt immobile posture or sleep compared to the control group. In addition, the disease control group had a statistically significant higher total time spent on personal hygiene compared to the normal control group (*p* < 0.01). The quercetin-treated groups were less engaged in grooming behaviors compared to the disease control group.

### 3.2. Effect of Quercetin on NTG-Induced Light-Aversive

**Figure 4:**
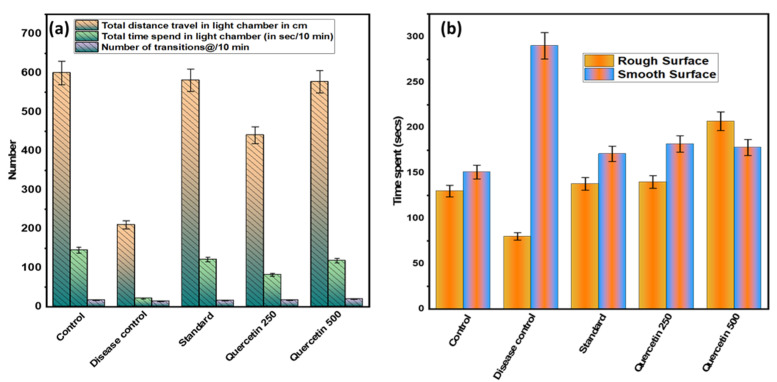
(**a**) Effect of quercetin on NTG-induced light-aversive behavior; (**b**) effect of quercetin on NTG-induced spontaneous tactile allodynia behavior.

After 90 min of NTG administration, the mildly aversive behavior was recorded (see Figure 4a). Statistically, the total distance traveled by the disease control group in the light chamber was minimal and significant (*p* > 0.001) compared to the normal control group. In addition, the disease control group spent the least total time in the light chamber compared to the normal control group (*p* < 0.01). It was found that the quercetin-treated groups spent statistically significantly more time in the light chamber than the disease control group. In addition, the number of 10 min transitions between groups was found to be not statistically significant.

### 3.3. Effect of Quercetin on NTG-Induced Spontaneous Tactile Allodynia Behavior

The disease control group spent significantly (*p* < 0.001) more time on the smooth surface analyzed for 5 min than the normal control group (see Figure 4b). The quercetin-treated groups were more involved and spent more time on a rough surface compared to the control group. However, on smooth surfaces, the quercetin groups were less involved and spent less time than the control group. Rats in the disease control group spent more time on the edges and corners of the smooth surface compared to the normal control group.

### 3.4. Effect of Quercetin on NTG-Induced Depression or Anxiety in Rats 

**Figure 5:**
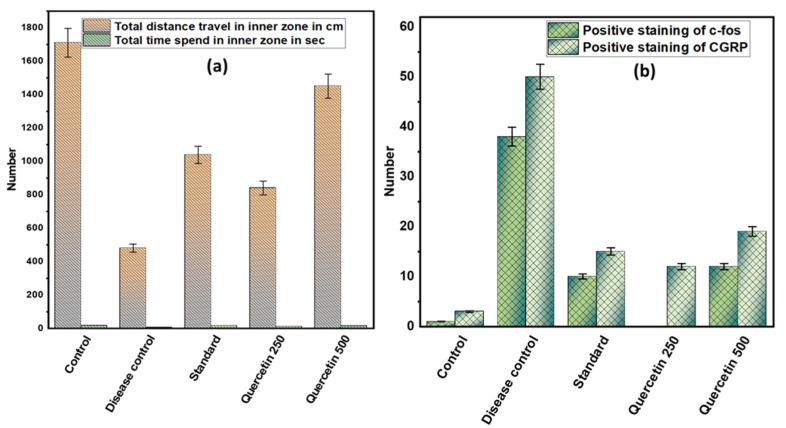
(**a**) Effect of quercetin on NTG-induced depression or anxiety in rats; (**b**) effect of quercetin on c-fos and CGRP immunoreactive cells in the TNC in rats.

Rats in the disease control group covered shorter distances in an open field maze compared to the control group (*p* < 0.001). Quercetin 500 mg/kg effectively increased the movement of rats in the open field maze (*p* < 0.01). However, the time spent by the rats in the inner zone of the maze did not differ significantly among the different groups. The rats in the disease control group spent more time in the corners of the outer zone than the control group. However, the rats in the quercetin 500 mg/kg group spent significantly more time in both the outer and inner zones (see Figure 5a).

### 3.5. Effect of Quercetin on C-Fos and CGRP Immunoreactive Cells in the TNC in Rats

The number of immunoreactive c-fos cells in TNC was higher in the disease control group than in the normal control group (*p* < 0.001). However, in the quercetin group, the number of immunoreactive c-fos cells in TNCs was reduced. Moreover, in the disease control group, the amount of positive staining of immunoreactive CGRP cells in TNC increased compared to the normal control group. However, in the 500 mg/kg quercetin group, the number of immunoreactive positive CGRP cells in TNC was significantly reduced (see Figure 5b).

## 4. Discussion

Migraine is a sapping illness. Its primary features are recurrent severe headaches associated with sounds or light, vomiting, dizziness, etc. It is a common and multifaceted neurological disorder that affects approximately 18% of women and 6–7% of men. [1] Migraine is promoted by both environmental and genetic factors. There are several options for the treatment of migraine or any form of primary headache [32]. In the present study, quercetin was selected for the treatment of migraine pain and associated behavioral changes in NTG-induced migraine. 

Some recent reports have claimed that NTG administration can induce behavioral changes such as head scratching, increased cage climbing, body shaking, etc. [33]. These behavioral changes persisted for 2–3 h after NTG injection and can be easily analyzed manually [25]. In the NTG-induced migraine model, rats showed more resting, grooming, and freezing behaviors and decreased activities of exploration and locomotion behaviors [34]. In general, migraineurs experience impairments in daily physical activity along with irritation during migraine pain. Cutaneous allodynia can increase grooming behavior in rats and is a common symptom in migraine attacks [35]. Therefore, recent experimental studies have shown that NTG-induced migraine is clinically applicable in rats due to the translational ability of behavioral endpoints [36]. 

Traditionally, quercetin has been used to relieve various types of pain and as an anti-inflammatory agent. In addition, preclinical studies with quercetin showed a good analgesic effect until the effect on behavior was not analyzed. The results showed that pretreatment with quercetin improved the frequency and total time of rearing and sniffing during exploratory behavior. The rats’ locomotor behavior also changed, and the frequency and total time of walking increased. One report found allodynia of approximately 63% in migraine patients, mimicking tactile allodynia in the migraine rat model. Numerous pharmacological reports have found that assessment of allodynia is a useful parameter for neuropathic pain in animals [37]. However, the assessment of tactile allodynia was performed using a rough–smooth apparatus in an NTG-induced migraine model. Our results showed that quercetin reduced the total time spent on the smooth surface during the rough/smooth test, suggesting that quercetin may reduce tactile allodynia [38]. In addition, pretreatment with quercetin reduced the total time spent in an immobile or resting state and the frequency of grooming (especially on the face). Thus, the results clearly demonstrate that quercetin can increase physical activity and reduce grooming behavior. In addition, the antimigraine effect and beneficial role of quercetin were found to be dose dependent. The efficacy of quercetin is evidenced by a reduction in locomotor behavior and a reduction in walking distances in the outdoor trials used to assess depressive or anxiety behaviors and quercetin. A somber or depressed rat tends to stay in a safe place when exposed to a new environment [39,40]. Consequently, pretreatment with quercetin increased the total distance traveled in the open-air environment. Depression or anxiety were comorbidities associated with chronic migraine. In addition, quercetin was reported to be an effective antidepressant [40]. Quercetin showed antidepressant effects via multiple targets, such as inhibition of oxidative stress and monoamine oxidase activity, protection of the neuronal system, modulation of the hypothalamic–pituitary–adrenal system, and an increase in the expression of neurotrophic factors [41]. 

In addition, quercetin-treated rats spent more time in the light chamber and also traveled a longer total distance in the light chamber. Photophobia is a common symptom in migraine, and this symptom was similarly induced by light-shy behavior in the light–dark box [42]. Quercetin reduced light-avoidance behavior, suggesting that quercetin alleviates the symptoms of photophobia. All these behavioral results suggest that pretreatment with quercetin may alleviate the behavioral changes associated with migraine pain induced by NTG [43].

Quercetin has analgesic and anti-inflammatory effects, probably due to the inhibition of prostaglandin production. [44]. In another study, quercetin showed efficacy in lipopolysaccharide-induced sepsis in mice by inhibiting endogenous inflammatory factors [26]. In addition, quercetin (0.15 μmol) significantly increases interleukin secretion and inhibits xylene-induced auricular swelling in rats, which showed anti-inflammatory activity [45]. In addition to its anti-inflammatory effect, quercetin also showed significant analgesic effect. Quercetin inhibited the nociceptive response, pain score, and thermal and mechanical pain sensitivity in spontaneous pain induced by bee venom [46]. The induced analgesic effect could be due to the blocking of pro-inflammatory factors such as prostaglandin (PGE2), leukotriene, etc. Quercetin inhibits the hyperalgesia induced in the sciatic nerve injury model [47]. NTG is a donor of nitric oxide (NO), which induces hyperalgesia or migraine-like painful and non-painful behavior in rats by activating trigeminal sensory nerve (TSN) fibers for 2 to 4 h. In addition, nitric oxide (NO) is a potent proinflammatory agent that plays a role in neuropeptide release or alteration and immune activation [48]. In a recent study [46], quercetin was found to have anti-inflammatory effects by regulating nicotinic acetylcholine receptors and reducing the release of pro-inflammatory cytokines in rats with ischemia-reperfusion injury in the brain.

Common hypotheses suggest that migraine-specific triggers are responsible for the initial brain dysfunction, which then leads to dilatation of cranial blood vessels innervated by the TSN fiber [25]. These dilated blood vessels allow the TSN fibers to become active, which in turn triggers a pain response that travels from the brainstem to higher brain regions [49]. This step triggers the release of vasoactive peptides such as CGRP, substance P, NO, and C-fos from TSN fibers. These vasoactive peptides cause vasodilation, which in turn leads to neurogenic inflammation, leakage of blood vessels, and degranulation of mast cells. CGRP and C-fos are thought to play an important role in the development and maintenance of headache, central sensitization, and allodynia, which are hallmarks of migraine disease pathogenesis [50]. Expression of C-fos is both a marker of cephalic nociception and a representation of neuronal activity in the central nociceptive pathway [31]. There is a correlation between the expression of c-fos and CGRP and the induction of hyperalgesia and allodynia by NTG [51]. According to the results of the current study, the number of immunoreactive CGRP and C-fos cells increased in the TSN fibers of the NTG-induced migraine rat model. However, pretreatment with quercetin attenuated this increase in cell number. In addition, CGRP receptor antagonists, such as tecagepant and olcegepant, were found to be effective and protective in migraine [52,53]. Our results are consistent with those of the CGRP receptor antagonist treatment study and suggest that pretreatment with quercetin is beneficial in migraine headache. 

Therefore, it can be concluded that quercetin has a protective effect in migraine pain through its analgesic effect and inhibition of inflammatory factors such as nitric oxide, proinflammatory cytokines, CGRP, C-fos, and so on.

## 5. Conclusions

Pretreatment with quercetin can improve locomotor, exploratory, resting, grooming, freezing, light-shy behavior, and allodynia in the NTG-induced migraine rat model. All these results suggest that quercetin can be used in the treatment of behavioral disorders and migraine pain. Mechanistically, quercetin reduces CGRP, immunoreactive C-fos cells, and other inflammatory factors involved in the development of migraine headaches. Nevertheless, there are several mechanisms and procedural limitations that we will need to overcome in further experiments to determine the exact role of quercetin in supporting the study.

## Figures and Tables

**Table 1:** Grouping of animals.

Groups	Subjects	Treatment Given
Group I	Normal control	0.9% saline
Group II	Disease control	NTG 10 mg/kg/I.P.only
Group III	Standard	NTG 10 mg/kg/I.P.only + sumatriptan 50 mg/kg oral
Group IV	Treatment I	NTG 10 mg/kg/I.P.only + Quercetin 250 mg/kg oral
Group V	Treatment II	NTG 10 mg/kg/I.P.only + Quercetin 500 mg/kg oral

NTG—nitroglycerin; I.P. Intraperitoneal.

## Data Availability

The data presented in this study are available on request from the corresponding author.
